# DNA Methylation Biomarkers Predict Objective Responses to PD-1/PD-L1 Inhibition Blockade

**DOI:** 10.3389/fgene.2019.00724

**Published:** 2019-08-16

**Authors:** Gang Xue, Ze-Jia Cui, Xiong-Hui Zhou, Yue-Xing Zhu, Ying Chen, Feng-Ji Liang, Da-Nian Tang, Bing-Yang Huang, Hong-Yu Zhang, Zhi-Huang Hu, Xi-Yu Yuan, Jianghui Xiong

**Affiliations:** ^1^SPACEnter Space Science and Technology Institute, Shenzhen, China; ^2^College of Informatics, Huazhong Agricultural University, Wuhan, China; ^3^State Key Laboratory of Space Medicine Fundamentals and Application, China Astronaut Research and Training Center, Beijing, China; ^4^Gastro-Intestinal Surgery Department, Beijing Hospital, Beijing, China; ^5^Department of Cardiothoracic Surgery, Strategic Support Force Medical Center of PLA. No. 9, Beijing, China; ^6^Department of Medical Oncology, Fudan University Shanghai Cancer Center, Shanghai, China; ^7^Department of General Surgery, Dongguan People’s Hospital affiliated to Southern Medical University, Dongguan, China

**Keywords:** PD-1/PD-L1 inhibition therapy, objective response rate, DNA methylation, biomarkers, Lasso model

## Abstract

Immune checkpoint inhibitor (ICI) treatment could bring long-lasting clinical benefits to patients with metastatic cancer. However, only a small proportion of patients respond to PD-1/PD-L1 blockade, so predictive biomarkers are needed. Here, based on DNA methylation profiles and the objective response rates (ORRs) of PD-1/PD-L1 inhibition therapy, we identified 269 CpG sites and developed an initial CpG-based model by Lasso to predict ORRs. Notably, as measured by the area under the receiver operating characteristic curve (AUC), our model can produce better performance (AUC = 0.92) than both a model based on tumor mutational burden (TMB) (AUC = 0.77) and a previously reported TMB model (AUC = 0.71). In addition, most CpGs also have additional synergies with TMB, which can achieve a higher prediction accuracy when joined with TMB. Furthermore, we identified CpGs that are associated with TMB at the individual level. DNA methylation modules defined by protein networks, Kyoto Encylopedia of Genes and Genomes (KEGG) pathways, and ligand-receptor gene pairs are also associated with ORRs. This method suggested novel immuno-oncology targets that might be beneficial when combined with PD-1/PD-L1 blockade. Thus, DNA methylation studies might hold great potential for individualized PD1/PD-L1 blockade or combinatory therapy.

## Introduction

Cancer immunotherapies have increasingly become a promising treatment strategy in the past few years. These therapies are designed to help the immune system identify and destroy cancer cells by targeting immune checkpoints such as programmed cell death protein 1 (PD-1) and its ligand (PD-L1) (Mahoney et al., 2015). PD-1 is expressed on the surface of activated T lymphocyte cells, and its major role is to inhibit T cell activation by binding to the PD-L1 ligand on cancer cells, leading to immune suppression (Medina and Adams, 2016). A number of immune checkpoint-modulating drugs that target PD-1/PD-L1 have shown remarkable clinical benefits in multiple cancers. For instance, nivolumab and pembrolizumab, the first two monoclonal antibodies approved by the US Food and Drug Administration (FDA) (Prasad and Kaestner, 2017), have already been registered for treatment of malignant melanoma (MM), advanced non-small-cell lung cancer (NSCLC), urothelial cancer, renal cell cancer, and head and neck squamous cell cancer (HNSCC) (Motzer et al., 2015; Robert et al., 2015; Reck et al., 2016; Bellmunt et al., 2017; Forster and Devlin, 2018). These drugs act by influencing the interaction between PD-1 and PD-L1, whose unobstructed interaction will downregulate T cells, causing cancer cells to evade immune surveillance (Prasad and Kaestner, 2017).

Compared with conventional therapy, inhibitors of PD-1 or PD-L1 can induce long-lasting responses in patients with metastatic cancer, but only one fourth to one third of patients have objective responses to immune checkpoint blockade therapy (Schachter et al., 2016). Additionally, these treatments are costly and might have some associated toxicities (Schmidt, 2017). Therefore, it is important to accurately identify the applicable population. Currently, emerging primary biomarkers used in response to immunotherapy are PD-1/PD-L1 protein expression, microsatellite instability (MSI), and tumor mutational burden (TMB) (Topalian et al., 2016; Chalmers et al., 2017; Chang et al., 2017). However, obvious limitations exist among these biomarkers due to low efficacy, antibody discrepancy, sampling bias, and strict requirements for cancer tissue. Achieving accurate forecasts and guiding clinical treatment remain critical challenges (Johnson et al., 2016).

The abnormal epigenomic landscape is one of the hallmarks of tumor initiation and progression (Esteller, 2008; Tsai and Baylin, 2011). In particular, aberrant patterns of DNA methylation can alter chromatin structure and gene transcription without altering the DNA sequence (Bird, 2007); these patterns have been extensively studied. In mammals, DNA methylation is almost exclusively found in CpG dinucleotides (CpGs). Recent work has revealed that DNA methylation affects tumorigenesis by regulating the tumor microenvironment (Xiao et al., 2016; Zhang et al., 2017). There are a multitude of DNA methylation biomarkers for the prognosis, diagnosis, and response to treatment in several types of cancer (Rodriguez-Paredes and Esteller, 2011). Based on the above evidence, we hypothesize that DNA methylation signatures could act as reliable immune checkpoint blockade biomarkers.

Ideally, abundant tumor molecule profiles along with patient objective response rates of immune inhibitors can be used to train reliable multiple biomarkers. However, in reality, only a small number of samples have both types of data. Alternatively, the tumor profiles are probably not the same ones whose response rates are assessed. For example, the research of Yarchoan et al. (2017) assessed the relationship between the tumor mutational burden and the objective response rate of PD-1/PD-L1 inhibition by pooling the response data from the published studies and the tumor mutational burden for each tumor type, which was provided by Foundation Medicine (Chalmers et al., 2017), and their analysis was that the sequenced tumor specimens may be different from those whose clinical responses were evaluated (Yarchoan et al., 2017). Similar to their method, we collected a large amount of DNA methylation profiles from 18 cancer types in The Cancer Genome Atlas (TCGA, https://tcga-data.ncbi.nih.gov/tcga/) and corresponding objective response rates from the largest published studies. We calculated the correlations between CpG probes and response rates in the 18 cancer types and then used CpG probes that were significantly correlated with response rates to construct a model for predicting the objective response rate by the Lasso regression method. We proposed that, compared with the model of predicting the response rate with TMB, the method with the CpG signatures was more accurate. Next, we utilized multimethod detection to verify the reliability of the DNA methylation signatures as surrogate biomarkers to predict the objective response rate of PD-1/PD-L1 inhibition.

## Materials and Methods

### Data Availability

The objective response rate (ORR) data for PD-1/PD-L1 inhibitors were obtained from the study of Yarchoan et al. (2017), and the data sets of the samples of each cancer were retrieved from TCGA (https://tcga-data.ncbi.nih.gov/tcga/). Each data set contained DNA methylation profiles obtained by Illumina 450K methylation assays. According to the research of Yarchoan et al. (2017) and the cancer types of TCGA, 18 cancer types have validated both ORRs and 450K methylation array data. In this study, these 18 cancer data sets were analyzed (**Table 1**).

**Table 1:** Objective response rates (ORRs) collection of 18 cancer types.

Tumor types	Abbreviation	ORR (literature)
Adrenocortical carcinoma	ACC	0.06 (Le Tourneau et al., 2017)
Bladder urothelial carcinoma	BLCA	0.182 (Rosenberg et al., 2016; Apolo et al., 2017; Bellmunt et al., 2017; Powles et al., 2017; Sharma et al., 2017)
Breast invasive carcinoma	BRCA	0.052 (Dirix et al., 2018)
Cervical squamous cell carcinoma and endocervical adenocarcinoma	CESC	0.208 (Hollebecque et al., 2017)
Esophageal carcinoma	ESCA	0.112 (Chung et al., 2016; Fuchs et al., 2017)
Glioblastoma multiforme	GBM	0.08 (Reardon et al., 2017a; Reardon et al., 2017b)
Head and neck squamous cell carcinoma	HNSC	0.16 (Ferris et al., 2016; Bauml et al., 2017)
Kidney renal clear cell carcinoma	KIRC	0.25 (Motzer et al., 2015)
Liver hepatocellular carcinoma	LIHC	0.2 (El-Khoueiry et al., 2017; Wainberg et al., 2017)
Lung adenocarcinoma	LUAD	0.19 (Borghaei et al., 2015)
Lung squamous cell carcinoma	LUSC	0.2 (Brahmer et al., 2015)
Mesothelioma	MESO	0.167 (Scherpereel et al., 2017)
Ovarian serous cystadenocarcinoma	OV	0.097 (Brahmer et al., 2012; Hamanishi et al., 2015; Disis et al., 2016)
Pancreatic adenocarcinoma	PAAD	0 (Brahmer et al., 2012)
Sarcoma	SARC	0.11 (Dangelo et al., 2017; Tawbi et al., 2017)
Skin cutaneous melanoma	SKCM	0.387 (Larkin et al., 2015; Robert et al., 2015)
Uterine corpus endometrial carcinoma	UCEC	0.13 (Fleming et al., 2017)
Uveal melanoma	UVM	0.036 (Algazi et al., 2016)

For independent verification to assess the robustness of our model, we collected the 450K methylation array data of NSCLC from the NCBI Gene Expression Omnibus (GEO) (http://www.ncbi.nlm.nih.gov/geo/) under accession number GSE39279, which includes 444 patient samples.

To calculate the TMB of the 18 cancer types, 18 Mutation Annotation Format (MAF) files processed by MuSE (Fan et al., 2016) were downloaded from the GDC data portal (https://portal.gdc.cancer.gov/repository). The MAF files contained the somatic mutations of TCGA cohorts.

Three hundred twenty-four annotated KEGG pathways comprising 7,448 genes (Entrez Gene IDs) were retrieved from Kyoto Encylopedia of Genes and Genomes (KEGG) pathway database (https://www.genome.jp/kegg-bin/get_htext?hsa00001+3101). These data were used for pathway analysis.

A human protein-protein interaction (PPI) network was derived from the STRING database (STRING, http://www.string-db.org). The default score threshold of interactions is typically 400 (Franceschini et al., 2013). Therefore, interactions with scores lower than 400 were discarded. These PPIs were used to construct subnetworks for a given gene.

### Identification of CpG Probes Associated With ORR

We used the β values reported by the 450K Illumina platform for each probe as the methylation level measurement for the targeted CpG site. The range of the β value is from 0 (no methylation) to 1 (100% methylation). A higher β value indicates a higher DNA methylation level. Each CpG value in a cancer type was represented by the mean β values in the tumor samples; then, the Spearman’s rank correlation test was used to quantify the association strength between the methylation level of the CpGs and the ORRs of the 18 cancer types. Since Bonferroni adjustment for multiple comparisons of the ∼480,000 CpGs is too conservative, especially with the small sample size (18 cancer types) in our research, we used a less stringent threshold of P value ≤0.001 and an absolute value [Spearman’s rank correlation coefficient (Spearman’s rho)] = 0.7 to obtain reliable ORR-associated CpG signatures. The annotation of each CpG, such as CpG’s position in the genome and its corresponding gene, was derived from the GEO database (https://www.ncbi.nlm.nih.gov/geo/query/acc.cgi?acc=GPL13534).

### Defining Methylation Levels of Functional Modules Based on Entropy

At a wide range of genomic positions, the CpG signals do not conform to a normal distribution but are distributed in a nearly bimodal distribution. Thus, too much information would be lost when simply averaging the β values.

In information theory, concept entropy is the average rate at which information is produced by a stochastic source of data. When the data source has a lower probability value (i.e., when a low-probability event occurs), the event carries more “information” (“surprisal”) than when the source data have a higher probability value. The amount of information conveyed by each event defined in this way becomes a random variable whose expected value represents the information entropy. Generally, entropy refers to disorder or uncertainty. Here, we capture the methylation levels of various functional modules based on Shannon’s entropy, which is described as follows:

$$H=-\sum_{i=1}^{n}\left(p_{i}\ln\;p_{i}\right)$$

In this equation, pi is the β value of each CpG probe and n is the number of CpG probes within the functional modules (protein network, KEGG pathway, and ligand-receptor gene pairs). Likewise, we used the Spearman correlation test to quantify the strength of associations between each functional unit and the ORRs of 18 cancer types.

### Construction of the CpG-Based Lasso Regression Model

To predict the objection response rate of PD-1/PD-L1 inhibition with reliable CpG signatures for clinical applications, additional selection and model construction are necessary. The Lasso algorithm is used to perform the variable selection procedure by estimating linear regression coefficients by L1-constrained least squares. It minimizes the sum of squared residuals, which is affected by the sum of the absolute values of the coefficients being less than the constant. Because of this constraint, Lasso regression tends to produce some coefficients that are precisely 0. Finally, a robust and interpretable model can be given. The original linear regression model can be written as follows:

$$y=\alpha +\beta_{1}x_{1}+\beta_{2}x_{2}+\cdots +\beta_{p}x_{p}+\in$$

The Lasso estimates for the constant term (α) and the regression coefficient (β) are as follows:

$$\left(\hat{\alpha },\hat{\beta }\right)=\mathrm{argmin}\sum_{i=1}^{n}(y_{i}-\alpha_{i}-\sum_{j=1}^{p}\beta_{j}x_{\mathrm{ij}}{)}^{2},\mathrm{s.t.}\sum_{j=1}^{p}|\beta_{j}|\leq \lambda$$

Here, *y* represents the ORR values of 18 cancers, *x* represents the *β* values of CpG probes that are significantly associated with ORR, and λ is a nonnegative adjustment parameter that controls the amount of shrinkage. The determination of λ can be estimated using the cross-validated (CV) method proposed by Efron and Tibshirani in 1997 (Efron and Tibshirani, 1997). In this study, the Lasso function in MATLAB was used to fit the equation, and the CV was set to 10.

### Tumor Mutational Burden (TMB) Calculations

TMB is a measure of the number of somatic protein-coding base substitutions and insertion/deletion mutations occurring in a tumor specimen. To calculate the TMB, the total number of mutations counted is divided by the size of the genome examined. Here, we used 38Mb as the estimate of the exome size. The somatic mutations were counted from the MAF files of TCGA, and the tumor mutational burden for each patient was estimated as follows:

$$\mathrm{TMB}=\frac{n}{38}$$

In this equation, *n* is the total number of missense mutations of a patient.

The median TMB for each cancer type can then be estimated as follows:

$$\mathrm{Median}\;\mathrm{TMB}=\frac{N}{38}$$

In this equation, *N* is the median number of coding somatic missense mutations in a cancer type.

Next, in line with Yarchoan et al.’s work, a new linear correlation formula that evaluates the relationship between the TMB and ORR was constructed as follows:

ORR=0.0768*In(X)+0.1313

Here, *X* is the median TMB of each cancer type.

### Synergy Index Calculations

A synergy index (S) was calculated to determine the presence of the interactions of the β values of each ORR-associated CpG probe and TMB. The synergy index is equal to 1 (S = 1) in the absence of a synergistic interaction; in such a case, the joint effect of two predictive variables is equal to the sum of their independent effects (i.e., it is additive). A synergy index greater than 1 (S > 1) suggests the presence of a synergistic interaction; the observed joint effect is greater than that expected from the sum of the independent effects of the component variables (i.e., it is synergistic). Conversely, a synergy index less than 1 (S < 1) suggests an “antagonistic” effect or a negative interaction. Here, the synergy index was calculated via a logistic regression model.

## Results

### Identifying CpGs Associated With the Objective Response Rate (ORR) of PD-1/PD-L1 Inhibition Therapy

Based on Yarchoan et al.’s extensive literature searches, we obtained 18 cancer types for which validated ORRs and the 450K methylation array data are both available. From **Table 1**, we can observe that most ORRs of cancer types are less than 0.2.

We first performed Spearman’s rank correlation test to identify CpGs whose methylation level was associated with the ORRs of anti-PD-1/anti-PD-L1 therapy. We collected current global immuno-oncology targets as the gold standard to assess our result by the Kolmogorov–Smirnov (KS) test (Tang et al., 2018). The targets that were more enriched in high Spearman rank correlation coefficient (Spearman’s rho) ORR-associated genes exhibited a smaller P value (derived from the KS test), which indicated that our result was reliable (P value = 0.0249). At the threshold of an absolute value (Spearman’s rho) ≥0.7 and a P value ≤0.001, we identified 269 genome-wide significant CpGs corresponding to 191 genes (**Table 2** and **Supplementary Table S1**). Then, we investigated the number of CpGs enriched in these 191 genes. The more enriched, the more likely they can be considered marker genes of anti-PD-1/anti-PD-L1 therapy. We annotated the functions of the top enriched genes from the UniProt database (https://www.UniProt.org/) and the literature (**Table 3**). For example, HLA-E [human leukocyte antigen (HLA) class I histocompatibility antigen, alpha chain E] is the most enriched gene in our results, and some studies have indicated that HLA class I antigen expression can be utilized in select patients who may benefit from anti-PD-1/PD-L1-based immunotherapy (Sabbatino et al., 2016; Chowell et al., 2018). Therefore, we have reasons to infer that other enriched genes could also be considered potential markers for anti-PD-1/PD-L1 therapy.

**Table 2:** List of the top 10 ORR-associated CpGs.

CpG	Gene symbol	Chromosome	Genomic coordinate	Spearman’s rho	P value
cg02358190	MAST4	5	66187002	−0.92514	3.91E−08
cg04033580	C22orf45; UPB1	22	24891666	−0.8539	6.53E−06
cg13459303	TMEM176B; TMEM176A	7	1.5E+08	−0.82912	2.11E−05
cg24644201	CREB3L1	11	46299066	0.822922	2.74E−05
cg25626312	CREB3L1	11	46299204	0.81776	3.39E−05
cg03885527	PLIN2	9	19125654	−0.81363	4.00E−05
cg05690644	GDF6	8	97158015	−0.81053	4.52E−05
cg23393637		14	95513095	−0.81053	4.52E−05
cg26981651	RNF5; RNF5P1	6	32147670	−0.81053	4.52E−05

These are the top 10 ORR-associated CpGs, and the 269 ORR-associated CpGs corresponding to 191 genes are provided in **Supplementary Table S1**.

**Table 3:** Top enriched genes and function.

Gene symbol	CpGs count	Function
HLA-E	6	HLA-E has a very specialized role in cell recognition by natural killer cells (NK cells)
PLEC	4	Interlinks intermediate filaments with microtubules and microfilaments and anchors intermediate filaments to desmosomes or hemidesmosomes
HIVEP3	4	Plays a role of transcription factor; binds to recognition signal sequences for somatic recombination of immunoglobulin and T-cell receptor gene segments
FOXD2- AS1	4	lncRNA FOXD2-AS1 promotes NSCLC progression through Wnt/β-catenin signaling (Rong et al., 2017)
FOXD2	4	Probable transcription factor involved in embryogenesis and somatogenesis
CREB3L1	4	Transcription factor involved in unfolded protein response (UPR)

NSCLC, non-small-cell lung cancer.

**Figure 1:**
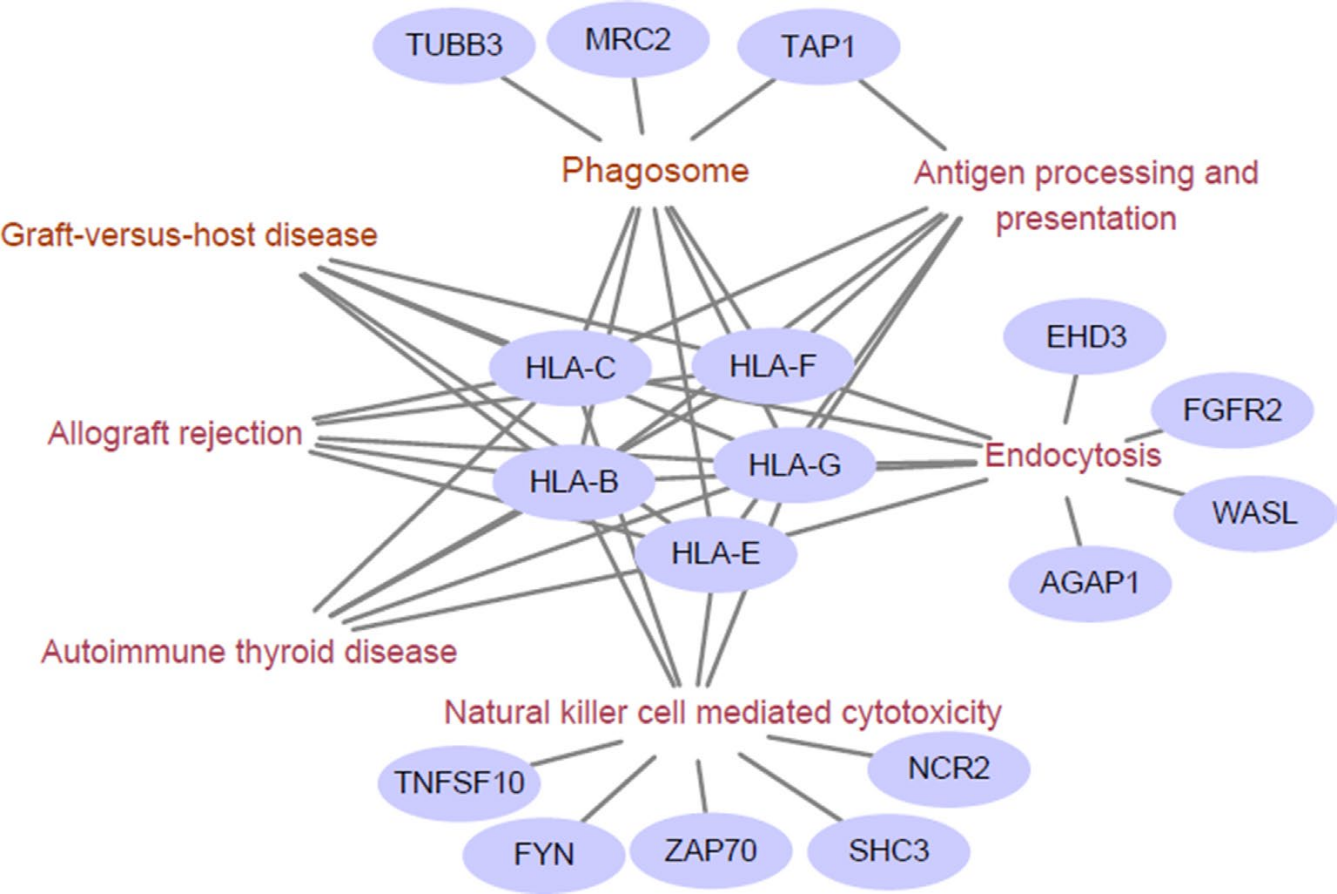
Enriched immunological pathway and genes. The words in wine represent the Kyoto Encylopedia of Genes and Genomes (KEGG) pathway, and the purple circles represent objective response rate (ORR)-associated genes enriched in the KEGG pathway. The HLA class I antigens (HLA-B, HLA-C, HLA-E, HLA-G, and HLA-F) are related to all these pathways.

We next examined the functional enrichment of these 191 genes using KEGG pathway analysis via cluster Profiler of R (Yu et al., 2012). Notably, we found that most of these genes were related to immunological KEGG pathways, such as antigen processing and presentation, natural killer (NK) cell-mediated cytotoxicity, and autoimmune thyroid disease (**Supplementary Table S2**). A recent study showed that the capacity of antigen presentation influences responses to checkpoint immunotherapy (Kvistborg and Yewdell, 2018), and tumor immunity is mediated mainly by NK cells (Ferrari de Andrade et al., 2018). Furthermore, we detected the signature genes that belong to multiple relevant immunological pathways. From **Figure 1**, we can clearly observe that HLA class I antigens are related to all these pathways, which highlights their importance in immunotherapy.

### Construction of the CpG-Based ORR Prediction Model by Lasso

To predict the objection response rate of PD-1/PD-L1 inhibition with reliable signatures for clinical applications, we used 269 CpGs that were obtained in the above section as initial variables to construct a model to predict ORR values by the Lasso algorithm.

First, we considered whether our CpG-based Lasso regression model method was generalized and practicable for predicting the ORRs of 18 cancer types. Therefore, we adopted a “leave-one-out cross validation” method to assess the feasibility of our model. Leave-one-out cross validation has been shown to give an almost unbiased estimator of the generalization properties of statistical models. Briefly, 17 cancer type-related data sets were used as training data sets for constructing the model, and the remaining data set was used as an independent data set. Then, we repeated this process 18 times to obtain the predicted ORRs of 18 cancer types. The Spearman’s rank correlation coefficient between the predicted and real ORRs was 0.75 (*P* value = 0.00029). This result indicated that our CpG-based Lasso regression model can be used to predict the ORRs of the 18 cancer types.

After the Lasso method was confirmed as being generalized and practicable, we used 269 CpGs and the ORR values of 18 cancer types to construct a prediction model by the Lasso algorithm. We chose the regression result when the mean square error (MSE) was minimum (MSE = 0.0042); there were eight CpG probe variables left: cg03749154, cg16051114 (DHCR24), cg04144714 (LYST, MIR1537), cg20395773 (ZBTB38), cg17484237 (HAVCR2), cg15006881 (GDF6), cg24644201 (CREB3L1), and cg13038847. The CpG-based prediction model is as follows:

$$\begin{array}{l} y_{\mathrm{ORR}}=0.793-0.526\times x_{\mathrm{cg}03749154}-0.0269\times x_{\mathrm{cg}16051114}s\\ \;\times x_{\mathrm{cg}04144714}+0.263\times x_{\mathrm{cg}20395773}-0.00086\times x_{\mathrm{cg}17484237}\\ \;-0.012\times x_{\mathrm{cg}15006881}+1.058\times x_{\mathrm{cg}24644201}-0.0603\\ \;\times x_{\mathrm{cg}13038847} \end{array}$$

**Figure 2:**
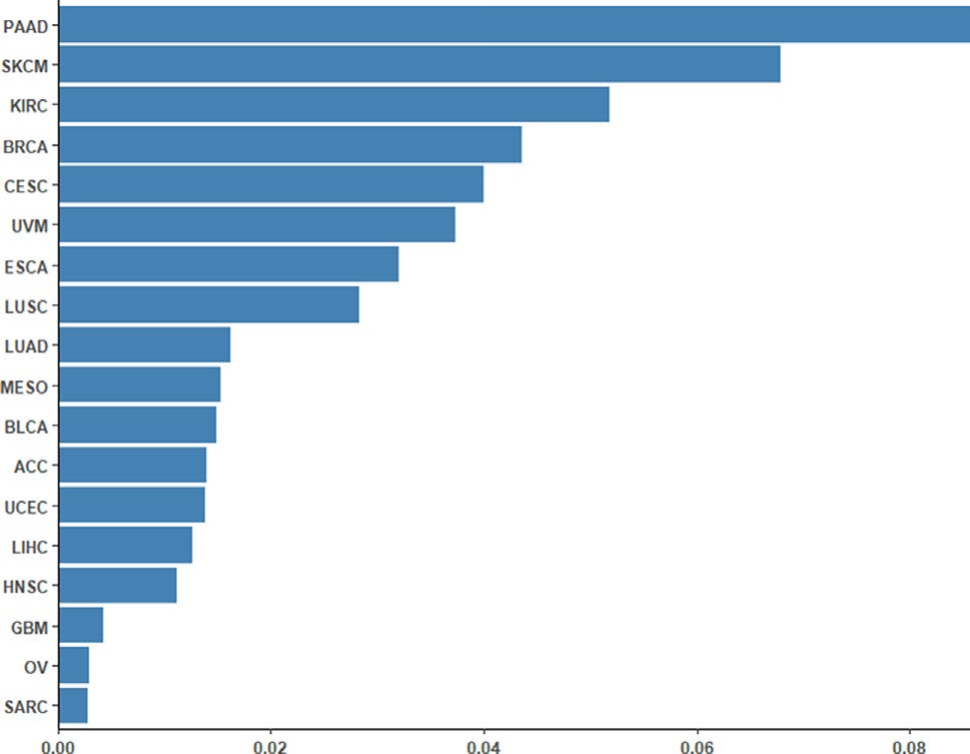
Differences between the predicted ORRs and true ORRs of 18 cancer types. Except for PAAD, SKCM, and KIRC, most of the ORRs of the cancer types could be predicted fairly robustly.

To assess the performance of the CpG-based prediction model for the 18 cancer types, we calculated the difference between predicted ORR values and true ones. As shown in **Figure 2**, except for PAAD, SKCM, and KIRC, the difference for other cancer types was very small. Moreover, to assess the robustness of our prediction model, we evaluated its performance in an independent sample of NSCLC from the GEO database. The ORR value of this independent data set was predicted to be 0.245, which was close to the real value of 0.2. This result further demonstrated that our model was accurate and robust.

### The CpG-Based Model Performs Better than the TMB-Based Model in ORR Prediction

In a study by Yarchoan et al., researchers evaluated the relationship between the TMB and the ORR. A linear correlation formula was constructed that can be used to make hypotheses with respect to the ORR rate in tumor types for which anti-PD-1/PD-L1 therapy has not been explored. Here, we compared the performance of our CpG-based model and our TMB-based model with respect to 18 cancer types.

**Figure 3:**
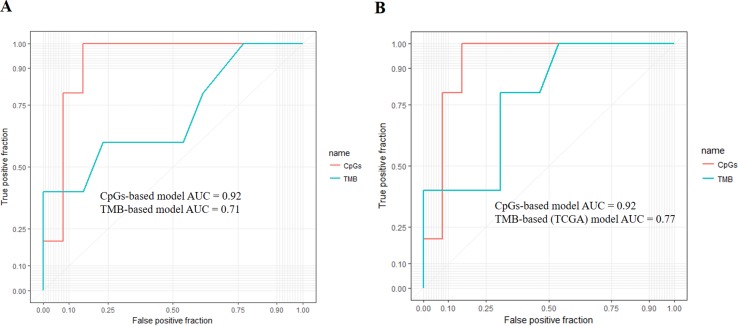
**(A)** Performance comparison of the CpG-based model and tumor mutational burden (TMB)-based model. The area under the receiver operating characteristic curve (AUC) scores of the CpG-based model and TMB-based model were 0.92 and 0.71, respectively, which indicated that our model had better performance. **(B)** Performance comparison of the CpG-based model and TMB-based model using The Cancer Genome Atlas (TCGA) samples. The AUC scores of the CpG-based model and TMB (TCGA)-based model were 0.92 and 0.77, respectively, which indicated that our model had better performance.

First, we adopted the root-mean-square error (RMSE), the mean absolute error (MAE), and Spearman correlations to compare the performance of the above two prediction models. As shown in **Table 4**, compared with the TMB model, the CpG-based model predicted ORR more accurately. Moreover, ROC curves were plotted to assess the sensitivities and specificities of these two models. As shown in **Figure 3A**, for the 18 cancer types, our model performs better than the TMB model in both sensitivity and specificity when 0.2 is used as a cutoff. The average area under the ROC curve (AUC) of the CpG-based model was 0.92, which was greater than the AUC of the TMB model, which was 0.71. For each cancer type, the performance evaluation criteria for the two models are compared to the actual ORR value of the cancer. The smaller the difference, the better the model effect. Except for CESC, HNSC, LUAD, and UVM, CpG-based model performs better than the TMB-based model in 14 cancer types. For the other four types of cancer, although CpG-based model is less powerful, our prediction is very close to the actual ORRs (**Supplementary Table S3**).

**Table 4:** Comparison of model performance.

Assessment index	CpG-based model	TMB-based model	TMB (TCGA)-based model
MAE	0.03	0.05	0.05
RMSE	0.04	0.07	0.06
Spearman correlation	0.93	0.58	0.69

TMB, tumor mutational burden; TCGA, The Cancer Genome Atlas; mean absolute error; RMSE, root-mean-square error.

To maintain data consistency between methylation and TMB, we recalculated the TMB of 18 cancer types from TCGA and constructed another linear correlation formula according to the results of Yarchoan et al.’s study. Then, we compared the performance of these two models as above (**Table 4**). As shown in **Figure 3B**, the AUC of our model was 0.92, which was greater than the AUC of the TMB model, which was 0.77. For each cancer type, except for ACC, BRCA, and UVM, CpG-based model performs better than the TMB (TCGA)-based model in 15 cancer types (**Supplementary Table S3**). This result further demonstrated that our CpG-based model was more accurate than the TMB-based model in ORR prediction.

### Combining CpGs and TMB in ORR Prediction

TMB and DNA methylation describe different aspects of immunotherapy work against cancer. TMB reflects the mutation signatures in cancer, while DNA methylation affects the tumor microenvironment (TME), which plays an important role in supporting cancer progression and tumor immunity (Zhang et al., 2017; Sanmamed and Chen, 2018). Therefore, after confirming that the methylation level of a few CpGs performs better at ORR prediction than TMB, we tried to combine these two types of information by computing the synergy index (*S*) between each ORR-related CpG and TMB.

A synergistic index greater than 1 (S > 1) suggests the presence of a synergistic interaction between TMB and ORR-associated CpGs, so combining TMB information could enhance the predictive ability of these CpGs. Furthermore, we investigated the top 10 CpGs that, in conjunction with TMB, have a synergistic effect (**Table 5**). Notably, TNFSF10 and HIVEP3, which were identified as being strongly correlated withPD-1/PD-L1 inhibition therapy in the previous section [rho (TNFSF10) = −0.75; rho (HIVEP3) = 0.75], also displayed strong synergy with TMB. This result indicated that these CpGs could also be applied jointly with TMB to achieve a higher prediction performance.

**Table 5:** List of the top 10 synergy sites.

CpG	Gene symbol	Synergy index
cg09248054	AGRN	37.17457358
cg22572614	TNFSF10	25.020374
cg23485436	KDM4B	24.3314227
cg25607920	HIVEP3	11.65557467
cg23902361	VAMP5	11.25511323
cg14116139		5.878589192
cg08405073	CCDC159	5.626048429
cg08405073	TMEM205	5.626048429
cg14615152	CSMD2	5.612506908
cg25577670	SVIL	5.466948003

### The Methylation Level of CpGs Is Associated With TMB at the Individual Level

**Figure 4:**
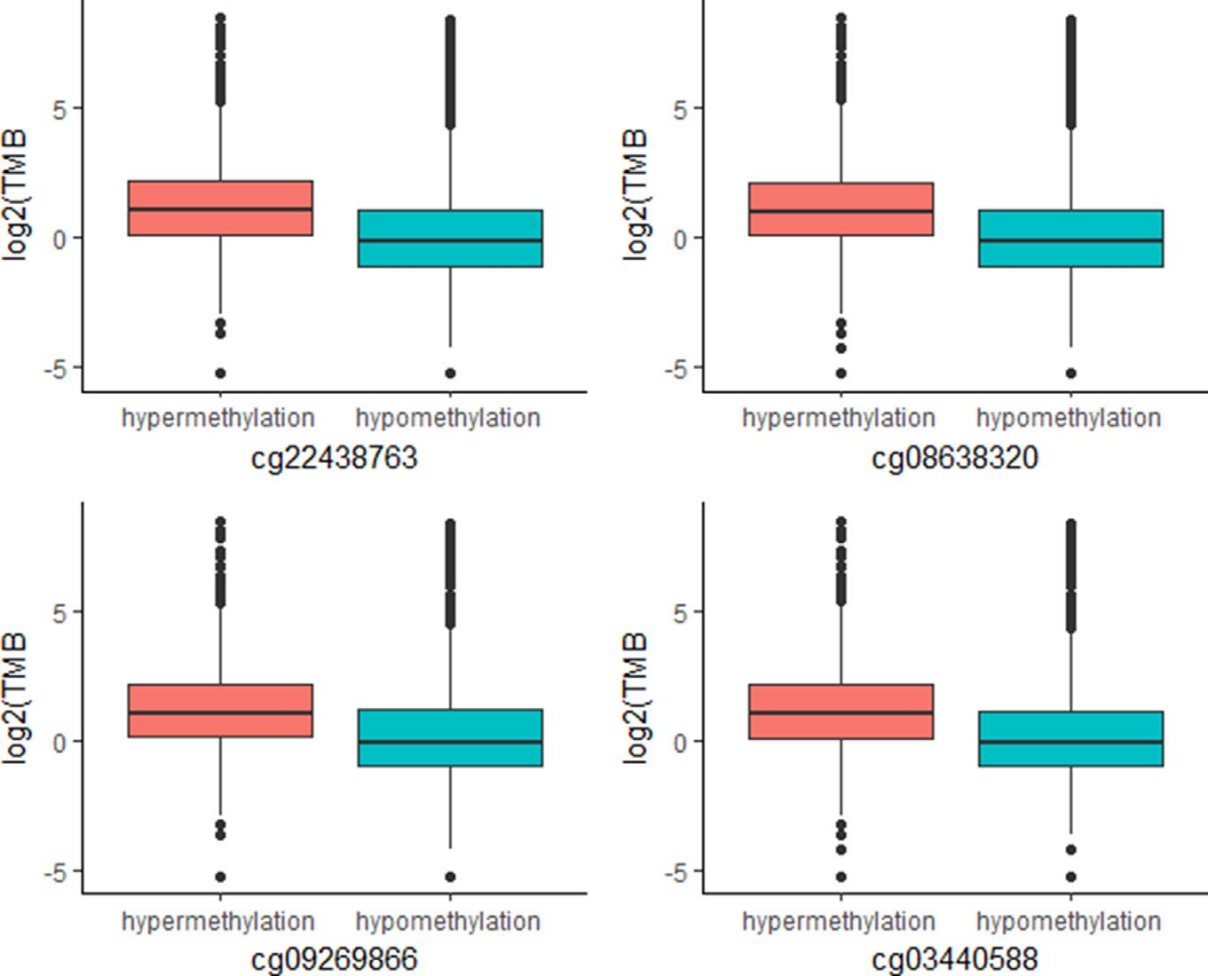
Plot of mutation burden in specimens with hypermethylation and specimens with hypermethylation of top TMB-associated CpGs (*n (specimens) = 5*,104).

The above work involved mainly identifying CpG signatures of ORRs; these signatures are meaningful and could be used to construct a model to predict ORRs at the cancer type level. However, for clinical applications, we are more concerned about whether these signatures could also work for individuals. Since TMB has become a relatively mature biomarker of sensitivity to immune check points in individuals, we identified these 269 CpG signatures whose methylation levels were associated with TMB at the individual level. Most of these CpGs were significantly associated with TMB with an FDR = 0.0001 (**Supplementary Table S4**). Moreover, we investigated the top associated CpGs and found that specimens with relatively high methylation levels of these CpGs are more likely to have relatively high TMB (**Figure 4**). These CpG signatures could also become biomarkers of PD-1/PD-L1 inhibition therapy for individual patients.

### Identification of DNA Methylation Modules Related to ORRs

A challenge of epigenetic studies is that DNA methylation changes can occur at a wide range of genomic positions, and their relationship between each single site and phenotype is not direct. A statistic to summarize the effects of environmental stimuli on gene regulation and the use of this feature to predict future medical events are highly desired. Here, we proposed a method to determine DNA methylation levels based on the entropy concept at different system levels, including protein networks, KEGG pathways, and ligand-receptor gene pairs, to represent coregulation units between two interactive cell types.

**Figure 5:**
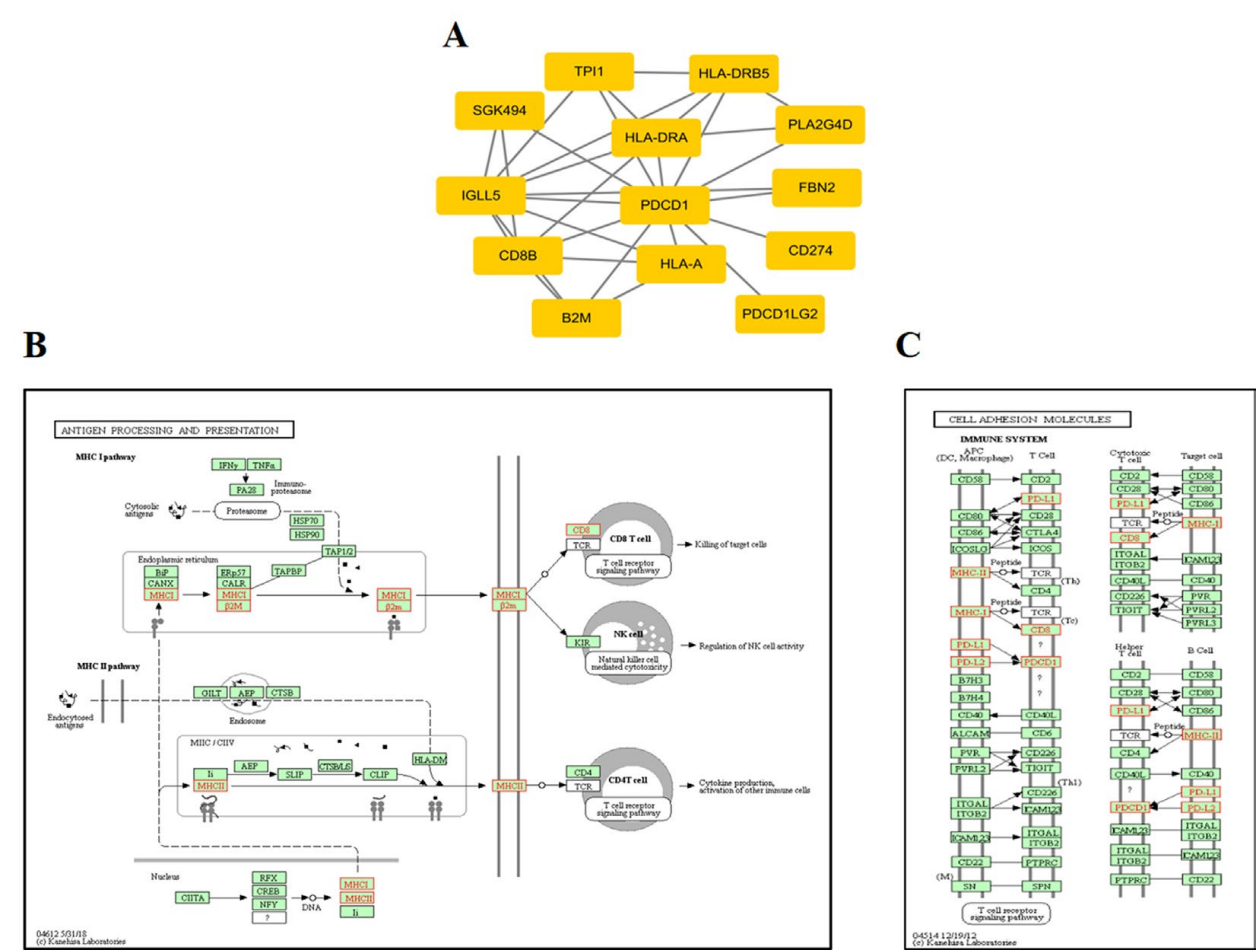
ORR-associated subnetwork map of KEGG pathways. **(A)** PDCD1 and its interacting genes in protein-protein interaction (PPI). **(B)** The pathway of antigen processing and presentation. **(C)** The pathway of cell adhesion molecules (CAMs); the golden yellow color represents the genes in the subnetwork of PDCD1.

At the protein network level, we found 787 subnetworks that were significantly associated with ORRs at a P value <0.05 threshold (**Supplementary Table S5**). Then, we focused on the subnetwork that contained PD-1 (PDCD1) and PD-L1 (CD274) (**Figure 5A**). This subnetwork is mainly involved in two pathways: antigen processing and presentation (**Figure 5B**) and cell adhesion molecules (**Figure 5C**). β2-Microglobulin (B2M) is a component of the HLA class I complex and functions in immunosurveillance. Carolina et al. reported that mutations in B2M could impair the correct formation of the HLA-I complex, which subsequently affects the response to anti-PD-1/anti-PD-L1 therapies (Pereira et al., 2017). Here, based on entropy to quantify the level of DNA methylation in a subnetwork, we obtained a similar observation. Except for PD-1/PD-L1, we should also pay more attention to the other subnetwork genes that may inspire new immunotherapies.

At the KEGG pathway level, 37 KEGG pathways were significantly associated with ORRs at a P value <0.05 threshold. Among them, several KEGG pathways were related to immune processes (**Supplementary Table S6**), such as the B cell receptor signaling pathway, the T cell receptor signaling pathway, natural killer cell-mediated cytotoxicity, and autoimmune thyroid disease. These results were consistent with the previously enriched pathway by 269 CpGs. Moreover, although other KEGG pathways are not directly related to immunotherapy, they are also meaningful. For instance, riboflavin metabolism is strongly significantly associated with ORRs; previous research has shown that metabolites of vitamin B represent a class of antigens that are represented by MHC class I-like molecules (MR1s) for mucosal-associated invariant T (MAIT) cell immunosurveillance (Kjernielsen et al., 2012). Therefore, our results may provide new insights into PD-1/PD-L1 inhibition therapy.

**Figure 6:**
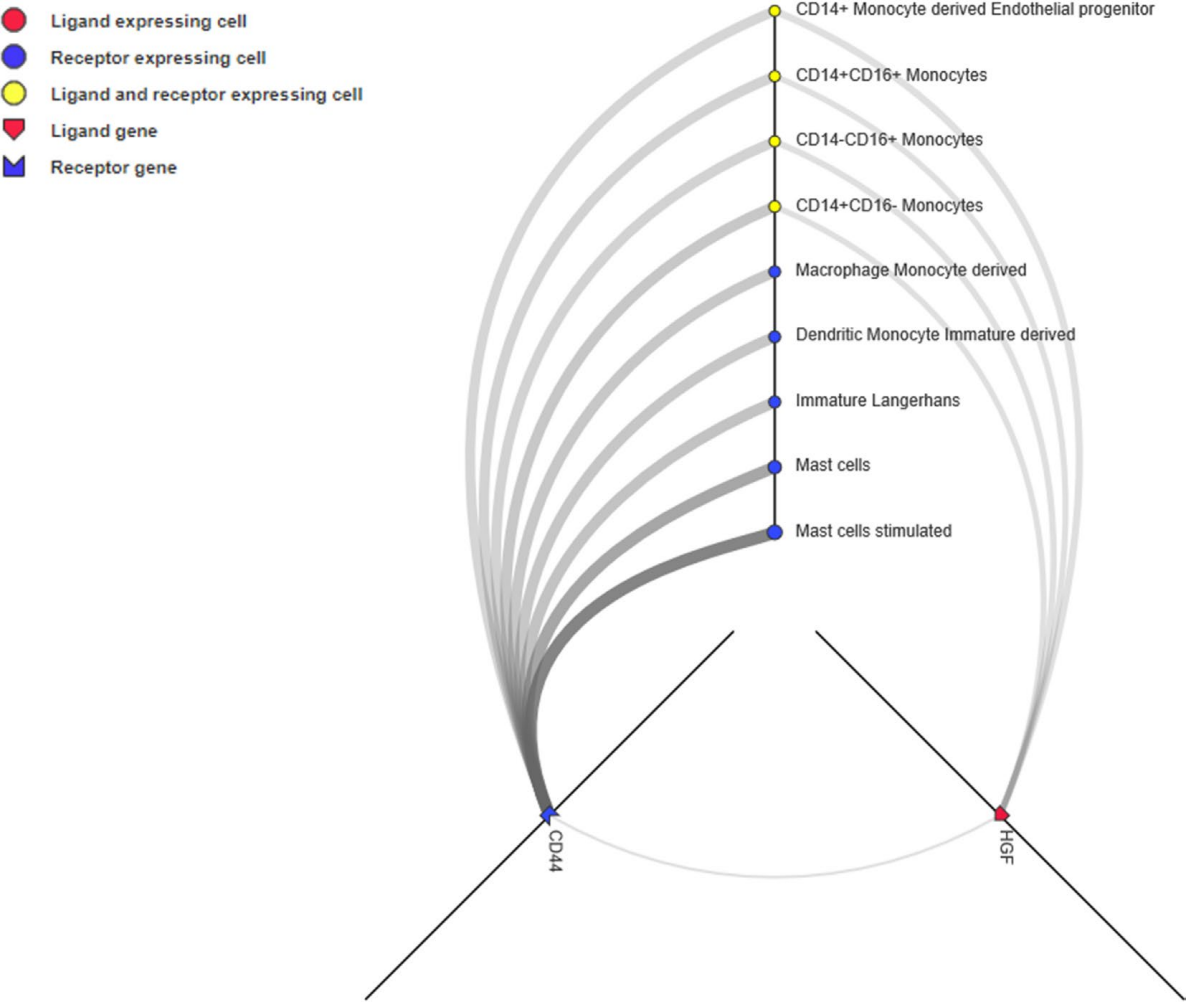
CD44-HGF signaling network interface. In this network, both CD44 and hepatocyte growth factor (HGF) were expressed in monocytes (≥10 TPM). The interface is available at http://fantom.gsc.riken.jp/5/suppl/Ramilowski_et_al_2015/.

Unlike the PPI network, which depicts the intracellular network, ligand-receptor mediated cell-to-cell communication across multiple cell types and tissues could inspire new immunotherapy techniques (Ramilowski et al., 2015). Ligands, receptors, and their interactions were retrieved from the CellPhoneDB (https://www.cellphonedb.org/) database. Including PD-1 and PDCD1, 103 ligand-receptor pairs were significantly associated with ORRs at a *P value ≤0.05 threshold* (**Supplementary Table S7**). The ligand-receptors of CD44 and HGF was the most significantly associated with ORR. Thus, we observed the cell-to-cell networks of CD44-HGF (**Figure 6**). We noted that CD44-HGF was expressed in monocytes at notable levels [≥10 Transcripts Per Kilobase Million (TPM)]. Although there is still no evidence that CD44-HGF affects the response to anti-PD-1/anti-PD-L1 therapies, a recent study identified types of immune cells known as classical monocytes (CD14+CD16–HLA-DRhi) in the peripheral blood as potential biomarkers for responses to anti-PD-1 immune checkpoint therapy in metastatic melanoma (Goswami et al., 2018).

From the above analyses, based on the entropy concept, we identified various functional modules associated with ORRs from the protein network, KEGG pathways, and ligand-receptor gene pairs. Some of these modules have been reported by other research groups, which confirmed the reliability of the DNA methylation signatures as surrogate biomarkers to predict the objective response rate of PD-1/PD-L1 inhibition.

## Discussion

Compared with conventional therapies, immune check point inhibitor treatments represented by PD-1/PD-L1 have shown remarkable clinical benefits (Yarchoan et al., 2017), but predictive biomarkers are needed. In this study, using DNA methylation profiles and the objective response rates (ORR) of 18 cancer types, we successfully identified 269 CpG signatures related to ORRs and developed an initial CpG-based objective response rate (ORR) prediction model by Lasso. We showed that these 269 CpG signatures (corresponding to 191 genes) can be considered potential immuno-oncology targets. Furthermore, the CpG-based ORR prediction model performed better than the TMB-based model. In the independent test of NSCLC data, our model also made accurate predictions. Moreover, we also identified CpGs that are associated with TMB at the individual level.

To further investigate the relationship between methylation and phenotype (i.e., ORR), we introduced a new method based on the entropy concept and identified various functional modules associated with ORR, from protein networks to KEGG pathways and ligand-receptor gene pairs, which may provide new insights into PD-1/PD-L1 inhibition therapy.

One limitation of our analysis is that the sequenced tumor samples were probably not the same for those whose ORRs were assessed, which would introduce deviation in our result. Matched clinical and genetic (i.e., DNA methylation profiles) data would help us develop a more robust and reliable model. The independent verification by bisulfite pyrosequencing of several most significant CpGs/genes can better demonstrate the accuracy of our conclusion. However, in the present study, we mainly focused on investigating the correlation between CpG methylation in genome and response to PD-1 or PD-L1 therapy and predicting ORR of cancer based on methylation level of several CpG sites in the patients. Based on statistical analysis and the evidence from the literature, it should be sufficient to draw a conclusion that such DNA methylation studies hold great potential for individualized PD1/PD-L1 blockade or combinatory therapy. Furthermore, CpG sites could also be applied jointly with other types of biomarkers, for instance, TMB, to achieve increased prediction performance to help oncologists select patients who are more likely to benefit from PD-1/PD-L1 inhibition therapy.

## Data Availability

All datasets analyzed for this study are included in the manuscript and the supplementary files.

## Author Contributions

JX, X-YY, Z-HH and H-YZ conceived and supervised the study. GX, Z-JC, X-HZ, and F-JL analyzed the data. GX and Z-JC wrote the manuscript. JX, Z-HH, YC, Y-XZ, D-NT, and B-YH made manuscript revisions.

## Funding

This research was funded by the grant from the National Instrumentation Program (No. 2013YQ190467), Shenzhen Science & Technology Program (JCYJ20151029154245758, CKFW2016082915204709), the Chinese Scientific and Technological Major Special Project (2012ZX09301003-002-003), Natural Science Foundation of China (91129708), the grant from State Key Lab of Space Medicine Fundamentals and Application (SMFA09A07, SMFA10A03, and SMFA13A04), Wu Jieping Medical Foundation (320.6750.18150) and the Fundamental Research Funds for the Central Universities (No. 2662017PY115).

## Conflict of Interest Statement

The authors declare that the research was conducted in the absence of any commercial or financial relationships that could be construed as a potential conflict of interest.

The handling Editor declared a shared affiliation, though no other collaboration, with one of the authors ZH.
